# Chemical and Thermal Characteristics of PEF-Pretreated Strawberries Dried by Various Methods

**DOI:** 10.3390/molecules29163924

**Published:** 2024-08-20

**Authors:** Aleksandra Matys, Małgorzata Nowacka, Dorota Witrowa-Rajchert, Artur Wiktor

**Affiliations:** Department of Food Engineering and Process Management, Institute of Food Sciences, Warsaw University of Life Sciences—SGGW, 02-787 Warsaw, Poland; malgorzata_nowacka@sggw.edu.pl (M.N.); artur_wiktor@sggw.edu.pl (A.W.)

**Keywords:** pulsed electric fields, electroporation, convective drying, infrared-convective drying, microwave-convective drying, vacuum drying, antioxidant capacity, TGA, DSC, FTIR

## Abstract

By increasing the permeability of the cell membrane of the treated material, pulsed electric fields (PEF) enhance the internal transport of various chemical substances. Changing the distribution of these components can modify the chemical and thermal properties of the given material. This study aimed to analyze the impact of PEF (1 kV/cm; 1 and 4 kJ/kg) applied to strawberries prior to drying by various methods (convective, infrared-convective, microwave-convective, and vacuum) on the chemical and thermal properties of the obtained dried materials (sugars content, total phenolic content, and antioxidant capacity (ABTS and DPPH assays); thermal properties (TGA and DSC); and molecular composition (FTIR)). PEF could have induced and/or enhanced sucrose inversion because, compared to untreated samples, PEF-pretreated samples were characterized by a lower share of sucrose in the total sugar content but a higher share of glucose and fructose. Reduced exposure to oxygen and decreased drying temperature during vacuum drying led to obtaining dried strawberries with the highest content of antioxidant compounds, which are sensitive to these factors. All PEF-pretreated dried strawberries exhibited a lower glass transition temperature (T_g_) than the untreated samples, which confirms the increased mobility of the system after the application of an electric field.

## 1. Introduction

The most frequently used drying method in the food industry is convective drying (CD) [1]. The mechanism of this method consists in providing the appropriate amount of thermal energy with a hot drying medium in order to evaporate the water placed inside the tissue of the dried material [2,3]. Heat is supplied to the food under atmospheric pressure, using heated air or heated surfaces. Then, the water vapor is removed with the air [4]. Convective dryers are characterized by simple construction, control, and handling, and relatively low investment costs. However, removing water from the material using hot air has many disadvantages, including, e.g., low thermal efficiency, significant energy consumption, and relatively long process duration, which negatively affect the product’s quality [5,6,7,8]. Subjecting the material to long-term exposure to oxygen and high temperature causes undesirable physical modifications of the product and leads to the destruction of sensitive bioactive ingredients [9]. Then, not only the sensory attractiveness of the material decreases but also its nutritional value [10].

One way to minimize the negative effects of convective drying is to combine it with other drying techniques during a single technological process, so-called hybrid drying (i.e., infrared-convective (IR-CD) or microwave-convective (MW-CD)) [9]. In the process of infrared drying, radiation reaches the surface of the dried material and penetrates it, and the radiation energy is converted into heat. The energy of infrared radiation is transferred from the heating element to the surface of the dried product without heating the surrounding air, which reduces energy losses. A combination of convective and infrared methods is more efficient than just electromagnetic radiation or hot air heating alone because it creates a synergy effect [11,12,13,14]. The main advantage of microwave drying is the internal generation of heat (in the entire volume of the product), enhancing the rate of removing water from the material [15]. The risk of uneven heat generation is a significant problem, as it may lead to burned spots on the sample [16,17]. During microwave-convective drying, heat is not transferred to the material, but it is generated within it. Microwaves can be used as an additional source of energy; therefore, lower processing temperatures can be used [18]. Additionally, microwave radiation is highly thermally efficient, which reduces energy demand and shortens drying time [4,19].

Another way is to remove the vast majority of air from the dryer chamber, which is drying under reduced pressure—vacuum drying (VD). Due to the fact that pressure reduction causes a simultaneous decrease in the boiling point of water, drying the material in such conditions also takes place at lower temperatures. Eliminating hot air as the drying agent reduces the risk of thermal and oxidative degradation of the dried material. Additionally, reduced heat demand makes it a less energy-consuming technique in comparison to traditional convective drying [4,20,21].

Each of the above-mentioned methods has its advantages and disadvantages. In general, the drying process is a very complex operation, because in its duration, mass and heat exchange occurs between the solid-like wet material and the drying agent. Additionally, energy can be supplied to the material being dried through radiation, convection, or conduction, as well as combinations thereof. The selection of the most optimal drying method should consider the characteristics of the matrix, which, however, can be modified in some way [4,22,23].

Plant tissue has a very complex structure, and each of its elements plays a specific role [24]. Apart from its involvement in transport and transpiration, it also constitutes the plant’s protective layer. The intact cell membrane of the material limits diffusion, which is the basis of certain processes, e.g., drying [25]. The notion of using pretreatments includes not only the inactivation of enzymes, which may contribute to product degradation, but also the inducing of changes in the cellular structure of the material, which would lead to increasing the drying rate, reducing energy consumption, and minimizing unfavorable changes occurring in the tissue during drying and, as a result, obtaining dried materials of appropriate quality [24,26].

Pulsed electric fields (PEF) is a novel nonthermal technique, the application of which as a preliminary treatment can lead to increased efficiency and/or reduced time of the subsequent processes: the cooking of, e.g., rice [27] and beef meat [28]; the drying of various fruits and vegetables [29,30,31,32,33,34]; the extraction of various chemical compounds [35,36,37]; the freezing–thawing of, e.g., duck meat [38]; the frying of, e.g., potato [39]; or the osmotic dehydration of, e.g., potato [40]. Such various possibilities of using PEF stem from the boosted permeability of the cell membrane resulting from the electroporation. The short-width electrical impulses supplied during this treatment cause the polarization of the treated cell membrane, leading to the above-mentioned electroporation—the formation of small pores within the tissue. Due to the increased porosity of the cell membrane, it is much easier to extract various intracellular components from the treated tissue, e.g., amino acids, antioxidants, lipids, minerals, pigments, polyunsaturated fatty acids, and proteins [41,42,43,44].

Strawberries are fruits whose consumption, due to their high content of antioxidants, fibers, and vitamins, brings many health benefits to the human body. Their popularity also results from their characteristic and desirable aroma, color, juicy texture, and sweetness [45]. Unfortunately, the seasonality and relatively short shelf life of these fruits pose significant challenges for the food industry, which is looking for an effective method of processing them [46]. So far, mainly the effect of pulsed electric fields on the physical properties of only freeze-dried strawberries has been studied [47,48]. In comparison to untreated samples, PEF-pretreated strawberries exhibit lower shrinkage, higher rehydration capacity, and lower firmness [47]. They were crispier and more uniform in terms of shape, they had bigger and more homogeneous pores, and their color was preserved better [48].

This research aimed to analyze the impact of the pulsed electric fields applied as the pretreatment of strawberries dried by various methods (convective, infrared-convective, microwave-convective, and vacuum) on the chemical and thermal properties of the obtained dried materials (sugars content, total phenolic content, antioxidant capacity, thermogravimetric properties, and molecular composition).

## 2. Results and Discussion

### 2.1. Sugars Content

**Figure 1:**
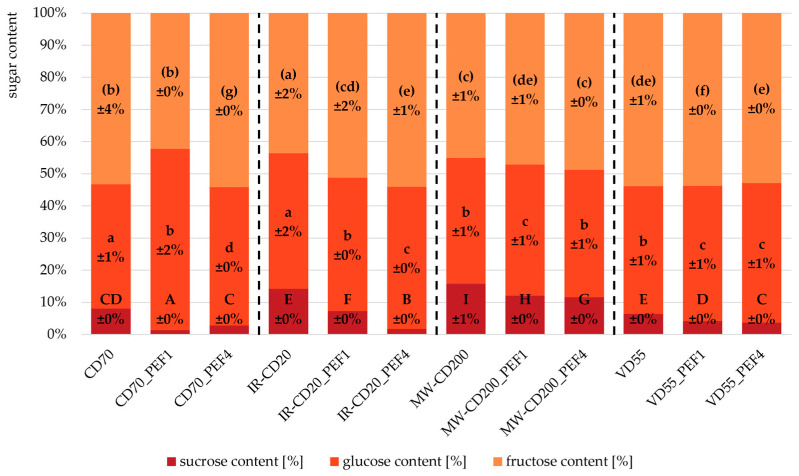
Share of sucrose, glucose, and fructose in the total sugar content in untreated and PEF-pretreated strawberries dried by various methods (CD—convective drying; IR-CD—infrared-convective drying; MW-CD—microwave-convective drying; VD—vacuum drying). The same letters, A–I; a–d; (a–g), indicate homogeneous groups (α = 0.05); ± standard deviation.

Figure 1 shows sugars content as the percentage share of individual sugars (sucrose, glucose, and fructose) in the total sugar content (TSC) in dried strawberries.

As one may notice, untreated samples (CD70, IR-CD20, MW-CD200, and VD55) were generally characterized by a higher share of sucrose in TSC than PEF-pretreated samples. This tendency was particularly visible in the case of strawberries dried using microwave-convective drying (MW-CD) and vacuum drying (VD) methods, where, additionally, along with an increase in the specific energy input from 1 to 4 kJ/kg, the share of sucrose in TSC decreased by approx. 18%. In turn, the application of PEF technology as a treatment prior to various drying methods generally led to a significant increase in the share of glucose and fructose in TSC in dried strawberries. As far as glucose is considered, this was most noticeable in the convective (CD) and infrared-convective (IR-CD) drying methods, in the case of which more intensely processed samples (CD70_PEF4 and IR-CD20_PEF4, respectively) were characterized by a 45 and 11% higher share of glucose in TSC from more mildly processed samples (CD70_PEF1 and IR-CD20_PEF1, respectively). With the increase in specific energy input from 1 to 4 kJ/kg, the share of fructose in TSC in IR-CD-dried strawberries increased by approx. 10%. The obtained results suggest that pulsed electric fields can cause and/or enhance sucrose inversion. In general, sucrose is a disaccharide that consists of fragments of two monosaccharides—glucose and fructose, linked together by an O-glycosidic bond. During the hydrolysis of sucrose, this bond is broken, which is accompanied by a phenomenon called sucrose inversion—a change in the sign of rotation. As a result, so-called invert sugar is created, which is a mixture of glucose and fructose molecules [49]. The causes of this phenomenon may be, for example, chemical factors (acids or bases activity), enzymatic factors (enzyme activity—sucrose inversion), or physical factors (high temperature activity) [50]. PEF can enhance the internal transport of various substances [51]. For example, the release of the sucrose invertase and the substrate of its enzymatic reaction, sucrose, makes it much easier to create an enzyme-substrate complex and, as a result, create the product of this hydrolysis reaction—invert sugar. Moreover, other researchers have observed an increase in the activity of the sucrose invertase enzyme, both native and denatured, as a result of applying the pulsed electric fields [52].

### 2.2. Total Phenolic Content (TPC)

**Figure 2:**
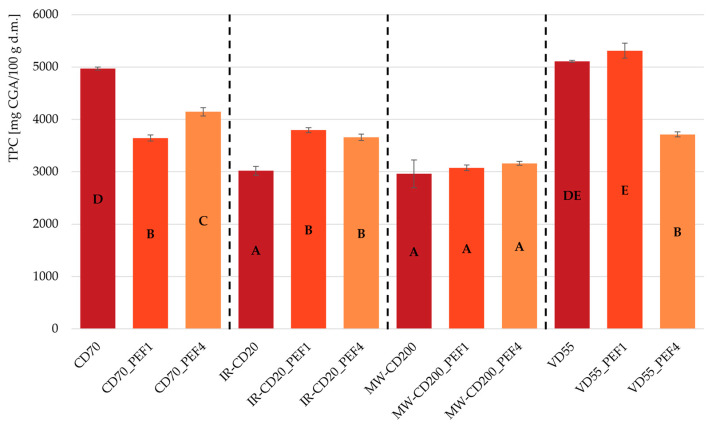
Total phenolic content (TPC) in untreated and PEF-pretreated strawberries dried by various methods (CD—convective drying; IR-CD—infrared-convective drying; MW-CD—microwave-convective drying; VD—vacuum drying). The same letters, A–E, indicate homogeneous groups (α = 0.05).

Total phenolic content in untreated and PEF-pretreated dried strawberries was expressed in milligrams of chlorogenic acid (CGA) per 100 g of dry matter, and it is presented in Figure 2. As one may observe, the retention of phenolic compounds in the dried strawberries depended on the drying method, the application of pulsed electric fields treatment, and the specific energy input. Considering only the drying method, the highest TPC was obtained by samples dried by VD. In general, the stability of polyphenols can be affected by many factors, e.g., ascorbic acid (Vc), enzyme, light, metal ions, nitrite salt, oxygen, pH, proteins, sulfur dioxide, and temperature [53]. So, better retention of phenolic compounds in VD-dried strawberries can be linked to reduced exposure to oxygen (lower pressure) and decreased drying temperature (lower boiling point of water) [4,20]. Sample VD55_PEF1 exhibited statistically identical total phenolic content as the untreated sample (*p* > 0.05), and this was the highest TPC value among all obtained samples. However, increasing the specific energy input from 1 to 4 kJ/kg resulted in a decrease in the retention of phenolic compounds, both in relation to the VD55 sample (untreated) and VD55_PEF1 sample (more mildly processed). The untreated CD-dried sample (CD70) had 27 and 17% higher TPC than samples CD70_PEF1 and CD70_PEF4, respectively. The main idea of applying PEF before drying is to loosen the treated matrix by boosting the permeability of its cell membrane. The decrease in the retention of phenolic compounds as a result of the PEF activity in the obtained dried strawberries may be related to the release of oxidizing enzymes (peroxidase, polyphenol oxidase) and/or their reaction substrates (phenols, flavonoids) from the tissue. As a result, the oxidation reactions of these compounds occur faster, leading to the formation of quinones, which then polymerize into brown pigments [54,55,56]. Moreover, the CD is carried out using hot air, which may enhance the oxidative and thermal degradation of phenolic compounds released by PEF. Increased temperature and the presence of oxygen, by causing the epimerization and autooxidation of polyphenols, may reduce their stability and bioactivity [53]. In general, samples dried by the IR-CD method exhibited lower TPC than strawberries dried by CD and VD. Heating of the material by infrared radiation is usually intense, but it takes place only on the surface of the sample. It can have an uneven effect, leading to local increases in the material temperature, and even lead to local burning [11,22]. Increased temperature has a negative effect on phenolic compounds [53]. PEF-pretreated IR-CD-dried samples exhibited TPC 21–26% higher than TPC in the untreated sample, which was dried in the same way. There were no statistically significant changes (*p* > 0.05) in TPC in strawberries dried by MW-CD (regardless of the use of PEF pretreatment and the differences in specific energy input). However, it is worth mentioning that the retention of phenolic compounds in MW-CD-dried strawberries was the lowest among the samples dried using the other methods. Assisting the drying process with microwave energy allows for the generation of heat in the entire volume of the material in the dryer. As a result, the temperature of such a sample may increase significantly [9,57,58,59]. As described above, temperature is one of the most crucial factors affecting the stability and bioavailability of polyphenols. Therefore, a significant increase in the temperature of the material during drying can result in the thermal degradation of phenolic compounds [53].

### 2.3. Antioxidant Capacity (ABTS and DPPH Assays)

**Figure 3:**
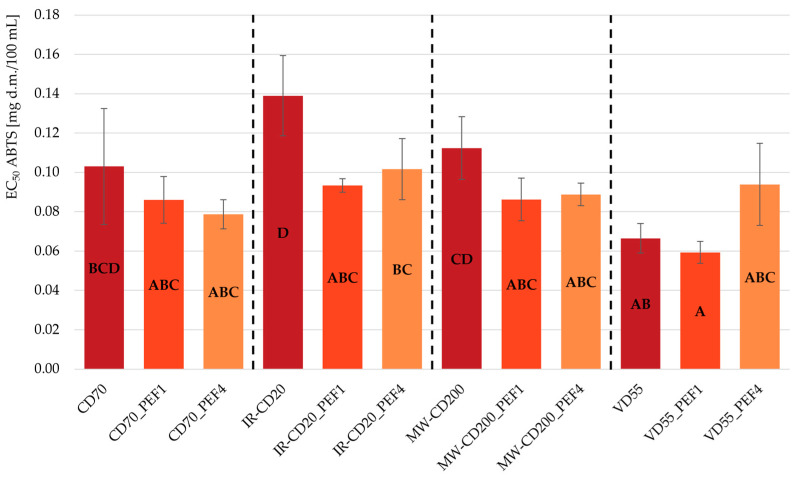
The EC_50_ values of scavenging ABTS^•+^ radicals cations with antioxidants contained in untreated and PEF-pretreated strawberries dried by various methods (CD—convective drying; IR-CD—infrared-convective drying; MW-CD—microwave-convective drying; VD—vacuum drying). The same letters, A–D, indicate homogeneous groups (α = 0.05).

**Figure 4:**
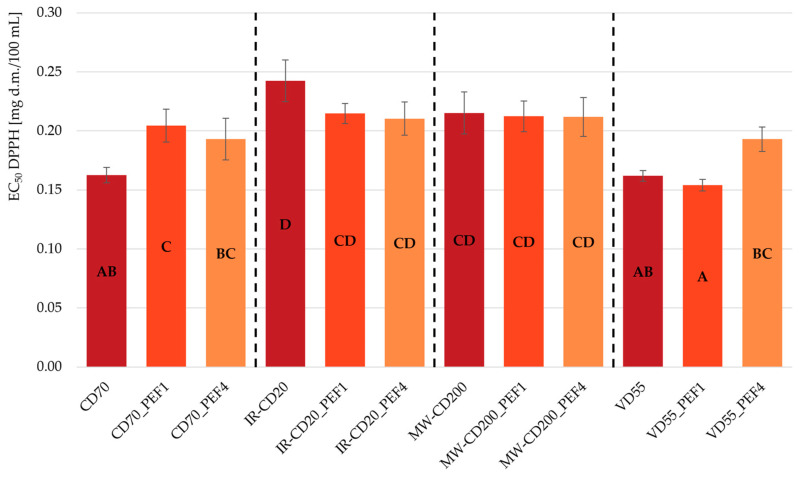
The EC_50_ values of scavenging DPPH^•^ radicals with antioxidants contained in untreated and PEF-pretreated strawberries dried by various methods (CD—convective drying; IR-CD—infrared-convective drying; MW-CD—microwave-convective drying; VD—vacuum drying). The same letters, A–D, indicate homogeneous groups (α = 0.05).

Figure 3 and Figure 4 show the antioxidant capacity of the obtained dried strawberries, measured in two ways: with the usage of ABTS^•+^ radical cations (Figure 3) and DPPH^•^ radicals (Figure 4). The results of both analyses were presented as EC_50_ coefficient—the sample extract concentration required for a 50% reduction in the initial amount of ABTS^•+^ radical cations and DPPH^•^ radicals. It means that the sample with the highest antioxidant properties is the sample with the lowest value of the EC_50_. Analyzing each drying method separately, regardless of the use of PEF pretreatment and the differences in specific energy input, showed that all three samples in CD, MW-CD, and VD were characterized by statistically identical antioxidant capacity determined using bluish-green-colored ABTS^•+^ radical cations (*p* > 0.05). In the case of IR-CD, the untreated sample showcased the worst antioxidant properties (the highest value of EC_50_—higher by 27–33% than those obtained for the PEF-pretreated samples dried in the same way). This is consistent with the result of the TPC analysis. In turn, when it comes to determining antioxidant capacity using deep purple DPPH^•^ radicals, no statistically significant differences were observed in the antioxidant properties of samples dried by IR-CD and MW-CD (*p* > 0.05). Taking into consideration only the samples within boundaries of the same drying methods, the CD70_PEF1 sample showed statistically lower antioxidant capacity than the untreated sample (CD70), and the same relation occurred between the VD55_PEF4 and VD55_PEF1 samples (*p* < 0.05).

### 2.4. Thermal Properties (TGA and DSC Analyses)

**Figure 5:**
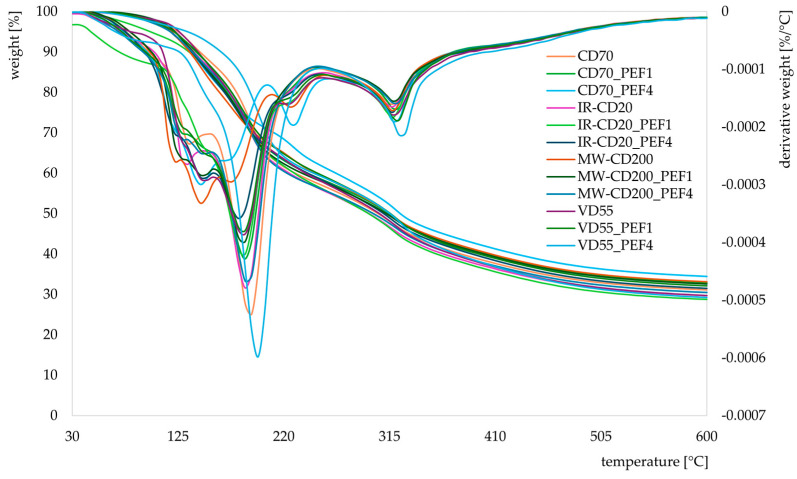
TG/DTG curves, obtained under a nitrogen atmosphere, of untreated and PEF-pretreated strawberries dried by various methods (CD—convective drying; IR-CD—infrared-convective drying; MW-CD—microwave-convective drying; VD—vacuum drying).

The thermal degradation behavior of the obtained dried strawberries is presented in Figure 5. All generated TG/DTG curves exhibited a similar pattern, with five stages of thermal decomposition. The first stage was at a temperature in the range of 30–110 °C (Table 1). During this stage, the weight loss of samples was between 2.5 and 6.1% and was related to water evaporation [60,61,62,63,64,65]. Even though the analyzed samples were dried materials, as mentioned earlier, they contained 0.04–0.11 g H_2_O/g d.m. In addition, they could absorb a small amount of water from the environment during preparation for analysis [66]. The second and third stages concerned temperatures in the range of 110–165 and 165–250 °C, respectively. At these stages, weight losses equaled 7.3–18.4 and 18.2–30.1%, respectively. It was at these stages that the first peaks appeared. The first one appeared at a temperature of 120.4–134.2 °C, the second one was observed at a temperature of 143.0–147.7 °C, the third one was recorded at a temperature of 171.2–195.5 °C, and the fourth one concerned a temperature of 226.7–228.8 °C. The first three ranges are the temperatures at which fructose, glucose, and sucrose are degraded [63], while the fourth one concerns hemicellulose. The fourth stage took place at a temperature in the range of 250–380 °C. During this stage, the weight loss of the samples equaled 16.8–20.2%, and the recorded peaks appeared at temperatures of 317.2–326.3 °C. Under such conditions, the decomposition of the polysaccharides, e.g., hemicellulose (200 to 320 °C), cellulose (280 to 400 °C), and lignin, takes place. The last stage was at a temperature in the range of 380–600 °C, at which further decomposition of lignocellulose occurs [62,64,67,68].

Food stability can be determined and predicted on the basis of the water activity (a_w_) and glass transition temperature (T_g_) that characterize that product [63]. Based on the obtained DSC thermograms of all the analyzed dried strawberries, their T_g_ was determined, the values of which are listed in Table 1. The glass transition of all samples occurred at temperatures ranging from −1.2 to 14 °C. As presented in Table 1, in the case of CD, MW-CD, and VD, all PEF-pretreated dried strawberries exhibited a lower temperature of glass transition than the untreated samples (CD70, MW-CD200, VD55), which confirms the increased mobility of the system after electric field application. The pores induced by PEF in the structure of the treated tissue caused an increase in free volume, intensification of mass transport, and redistribution of air and water. All those elements happen to increase the degree of mobility and thus reduce the glass transition temperature [69]. From the perspective of the stability of the dried materials and their desirability by consumers, a lower glass transition temperature of such products is unfavorable. Processing and storing dried materials at temperatures above T_g_ leads to a change from a more stable, brittle glassy state to a much less stable, elastic rubbery state. As a result of this phenomenon, the product becomes more susceptible to all kinds of chemical and physical changes, such as browning, loss of volatiles, moisture absorption, oxidation, or sugar crystallization. This may also result in the dried product softening and losing its characteristic crunchiness. Furthermore, materials in a rubbery state are more susceptible to microbial growth, contributing to reduced shelf life [70,71].

### 2.5. Fourier-Transform Infrared Spectroscopy (FTIR)

**Figure 6:**
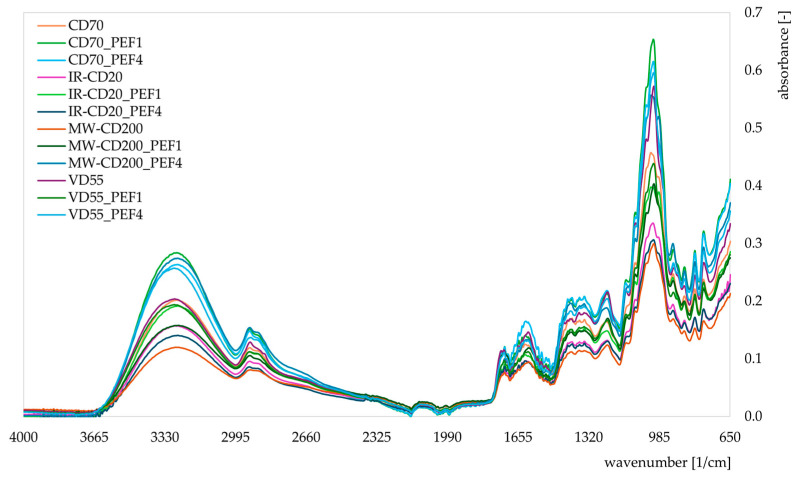
IR spectra of untreated and PEF-pretreated strawberries dried by various methods (CD—convective drying; IR-CD—infrared-convective drying; MW-CD—microwave-convective drying; VD—vacuum drying).

Figure 6 presents the infrared spectra (IR) obtained with the usage of FTIR analysis for untreated and PEF-pretreated dried strawberries. The main infrared peaks were observed in the range of the wavenumbers as follows: 3265–3283 1/cm (O-H symmetric stretch) [60], 2922–2929 1/cm (C-H asymmetric stretch of C-H_2_) [61,63,64,65], 1718–1722 1/cm (C=C stretch) [65], 1606–1621 1/cm (C=C aromatic ring) [60], 1230–1241 1/cm (O-C-H, C-C-H, and C-O-H bending) [65], 1013–1025 1/cm (C-O and C-C stretch, bending vibration of C-O-C) [60,63,65,72], 916–920 1/cm (C-O and C-C stretch) [65], 864 1/cm (C-H_3_ rocking) [65], 816 1/cm (C-H aromatic ring) [60], and 775 (bending vibration of C-H) [73]. The wavenumber range of 1000–1800 1/cm stands as the fingerprint region typical for pectin and other polysaccharides [60,74]. The analyzed samples all consisted of chemical compounds with similar functional groups, as indicated by a similar pattern of IR spectra [64]. However, the intensities of the obtained peaks in the recorded infrared spectra varied. Generally, higher peaks indicate a higher concentrations (contents) of particular molecular bonds in the material [75,76,77]. Samples with the highest peaks, especially at wavenumbers in the range of 3265–3283 1/cm and 1013–1025 1/cm, were CD70_PEF1, CD70_PEF4, MW-CD200_PEF4, and VD55_PEF4. This may indicate a higher content of alcohols, carboxylic acids, ethers, phenols, and some carbohydrates (O-H symmetric stretch, C-O and C-C stretch, bending vibration of C-O-C) in these samples [60,63,64]. In general, more intense peaks were recorded for PEF-pretreated strawberries than for untreated samples within the specific ranges of analyzed wavenumbers. This tendency was most noticeable in convective (CD) and microwave-convective (MW-CD) drying methods. The changes in IR spectra associated with PEF treatment could be correlated with the action of the electric field on the cell membranes and cellular components of the treated material, which could also involve structural changes in some compounds [78,79,80].

## 3. Materials and Methods

### 3.1. Materials

All technological and analytical procedures were performed on one and the same strawberry variety, “Rumba”, which was purchased from the local Polish producer (Warsaw, Poland) and then utilized on the same day. Only mature, similarly colored, and uninjured specimens were selected for the experiments. Surface pollution and soil residues were removed by rinsing the strawberries with tap water. The initial moisture content in fresh strawberries was equal to 11.53 g H_2_O/g d.m. (approx. 92.02%).

The chemical reagents necessary to perform all analyses were purchased from Sigma Aldrich (Saint Louis, MO, USA).

### 3.2. Technological Part

#### 3.2.1. Pretreatment—Pulsed Electric Fields (PEF)

In order to supply electric impulses, about 150 g of whole (uncut) strawberries were put into the treatment chamber, to which tap water was then added (σ = 718 μS/cm), filling it up to 1 kg of total input. The chamber with its entire input was inserted into a pulsed electric fields application device (PEFPilot™ Dual System, Elea Technology GmbH, Quakenbrück, Germany), which provides monopolar, rectangular pulses. Process parameters were set as follows: pulse frequency of 20 Hz, pulse width of 7 μs, and electrode voltage of 24 kV, which, combined with the distance between the two parallel stainless-steel electrodes of 24 cm, gives an electric field strength of 1 kV/cm. As the purpose was to provide specific energy inputs (W_s_) of 1 and 4 kJ/kg to the treated strawberries, the number of pulses necessary to achieve the intended goals was calculated from Equation (1), and it was equal to approximately 103 and 411, respectively.

(1) $$W_{s}=\frac{IUtn}{m}$$

where m is the mass of the total input of the treatment chamber (strawberries plus water) [kg], n is the number of electric pulses supplied to the treated strawberries [-], t is the pulse width [s], U is the electrode voltage [kV], and I is the current [A].

To ensure that the above-mentioned process parameters were effective in this type of matrix, the electrical conductivity of untreated, PEF-treated (W_s_ = 1 kJ/kg and W_s_ = 4 kJ/kg), and totally destroyed (W_s_ = 61 kJ/kg) strawberries was measured (pH/conductivity meter CPC-401, Elmetron, Zabrze, Poland). On the basis of those records, values of the cell disintegration index (Z) were calculated according to Equation (2). For specific energy inputs of 1 and 4 kJ/kg, Z was equal to 0.22 ± 0.03 and 0.38 ± 0.03, respectively.

(2) $$Z=\frac{\sigma_{t}-\sigma_{u}}{\sigma_{d}-\sigma_{u}}$$

where σ_u_, σ_d_, and σ_t_ are the electrical conductivities of untreated, totally destroyed, and PEF-treated strawberries, respectively [µS].

#### 3.2.2. Drying

Both untreated and PEF-pretreated strawberries were mechanically prepared for the drying process. After the removal of the calyxes, the fruits were cut into 5 mm thick slices. In this form, approximately 128 g of strawberry slices were directed to the dryer, where they were arranged in a monolayer on a tray area of approximately 0.035 m^2^, with a distance of approximately 1 cm between slices. Regardless of the drying method, each drying process was repeated three times and lasted until a constant weight was obtained, meaning that the mass was unchanged for at least 15 min.

##### Convective Drying (CD)

Convective drying was carried out in a laboratory convective dryer (Warsaw, Poland) at an air temperature of 70 °C and an air velocity of 1.5 m/s. The drying air flow was parallel to the dried strawberries. The tray load equaled approx. 1.25 kg/m^2^.

##### Infrared-Convective Drying (IR-CD)

Infrared-convective drying was conducted in a laboratory infrared-convective dryer (Warsaw, Poland), in which the distance between dried material and the source of radiation equaled 20 cm and an air velocity equaled 0.5 m/s. The source of infrared radiation was nine lamps arranged in three rows, each with a power of 175 W and a diameter of 125 mm. The total power of the infrared ray emitter equaled 7.875 kW/m^2^. The tray load equaled approx. 0.63 kg/m^2^.

##### Microwave-Convective Drying (MW-CD)

Microwave-convective drying was executed in a laboratory microwave-convective dryer (Promis-Tech Inc., Wroclaw, Poland) at a microwave power of 200 W, air temperature of 30 °C, and an air velocity of 2 m/s. The drying air flow was perpendicular to the dried strawberries. The microwave frequency equaled 2.45 GHz. The tray load equaled approx. 3.62 kg/m^2^.

##### Vacuum Drying (VD)

Vacuum drying was performed in a laboratory vacuum dryer (SPT-200, Conbest, Cracow, Poland) under a pressure of 4 kPa (boiling temperature of water equaled about 30 °C [81]) at an air temperature of 55 °C. The tray load equaled approx. 2.11 kg/m^2^.

### 3.3. Analytical Part

To stabilize the obtained dried strawberries, before starting the analytical examination, they were kept for a week in securely closed PET/AL/PE foil pouch packaging without access to external vapor, light, and gas.

#### 3.3.1. Dry Matter Content

The standard gravimetric method AOAC 920.15 [82] was used to quantify dry matter content (d.m.) in the fresh and the dried strawberries. Moisture content in the obtained dried strawberries was in the range of 0.04–0.11 g H_2_O/g d.m. (4.18–9.57%). Determination of the dry matter content was performed in triplicate.

#### 3.3.2. Sugars Content

The determination of the sugars content in the obtained dried strawberries was carried out according to the liquid chromatography method with refractive index detection [83]. The system used for the analysis consisted of a quadruple pump (Waters 515, Milford, MA, USA), an autosampler (Waters 717, Milford, MA, USA), a column thermostat, and a refractive index detector (Waters 2414, Milford, MA, USA). Separation was performed with the utilization of a 300 × 6.5 mm Waters Sugar Pak I column with a Sugar-Pak precolumn. First, 0.26 g of material previously ground with the use of an analytical mill (A11 basic, IKA^®^-Werke GmbH & Co. KG, Staufen, Germany) was extracted with 10 mL of ultrapure water at a temperature of 80 °C for 4 h. The prepared solution was filtered through a 0.45 µm PTFE syringe filter, and after that, it was dispensed directly into the system (volume of injection equaled 1 µL). The determination was carried out under isocratic conditions; the flow rate of the mobile phase (Milli-Q ultrapure water with 18.2 MΩcm, and at a temperature of 25 °C) was 0.6 mL/min, and the temperature of the column and the detector equaled 90 and 50 °C, respectively. Aiming to quantify sugars in obtained dried strawberries, prepared calibration curves for standards of sucrose, glucose, and fructose were used. The analysis of the sugars content was performed in duplicate.

#### 3.3.3. Extraction Procedure

In order to determine selected chemical compounds (phenolics and other antioxidants contained in the obtained dried strawberries), it was necessary to extract them from those solid-like matrices first. About 0.3 g of previously ground materials (analytical mill A11 basic, IKA^®^-Werke GmbH & Co. KG, Staufen, Germany) and 10 mL of solvent (ethanol-water solution, 80:20 mL/mL and 0.1 M hydrochloric acid, mixed in the ratio of 85:15 mL/mL) were put into 15 mL falcon tubes. Such prepared solutions were mixed for about 12 h with the usage of the multi-vortexer (Multi Reax, Heidolph Instruments, Schwabach, Germany), at room temperature (approx. 20 °C). After that process, the sample extracts were centrifuged at 4350 rpm for 2 min in a bench top centrifuge (MegaStar 600, VWR, Leuven, Belgium), and then they were dispensed into the 0.2 mL PCR tubes with flat caps. Two extracts were prepared from each sample.

#### 3.3.4. Total Phenolic Content (TPC)

The spectrophotometric method using Folin and Ciocalteu’s phenol reagent, proposed by Singleton et al. [84], was used to determine the total phenolic content in the dried strawberries. For that purpose, 10 µL of distilled water, 10 µL of sample extract prepared according to the procedure described in Section 3.3.3, and 40 µL of five-times-diluted Folin and Ciocalteu’s phenol reagent were dosed one by one into the 96-well plates. The reaction lasted 3 min, and it was finished by adding 250 µL of 7% sodium carbonate solution. Incubation was carried out in total darkness for 1 h, at room temperature (approx. 20 °C). A plate reader (Multiskan Sky, Thermo Electron Co., Waltham, MA, USA) was used to measure the absorbance of the prepared solutions at a wavelength set to 750 nm, in relation to a blank test (10 µL of solvent instead of 10 µL of sample extract). Chlorogenic acid served as the standard (concentration range of the calibration curve with the CGA: 0–100 µg/mL), so the results were presented as mg of chlorogenic acid (CGA) contained in 100 g of dry matter (d.m.) of the obtained dried strawberries. Each sample was tested four times (double analysis of each of the two sample extracts).

#### 3.3.5. Antioxidant Capacity (ABTS and DPPH Assays)

The reducing ability of antioxidants toward ABTS^•+^ radical cations and DPPH^•^ radicals was used to determine the antioxidant capacity of the obtained dried strawberries [85]. It was necessary to prepare stock solutions of ABTS and DPPH before starting the analyses [86]. For this purpose, 38.4 mg of 2,2′-azino-bis(3-ethylbenzothiazoline-6-sulfonate) and 6.6 mg of potassium persulfate were dissolved in 10 mL of distilled water (ABTS stock solution). To prepare the DPPH stock solution, 25 mg of 2,2-diphenyl-1-picrylhydrazyl was added into the 100 mL volumetric flask, which was then filled up to 100 mL using a methanol–water solution, 99:1 mL/mL. Both ABTS and DPPH stock solutions were stored under refrigerated conditions. In order to obtain working solutions, ABTS and DPPH stock solutions were diluted by an ethanol–water solution, 80:20 mL/mL, to a level that resulted in an absorbance of approx. 0.7 absorbance unit, measured in a 1 cm glass cuvette, at wavelengths set to 734 and 515 nm for ABTS and DPPH, respectively. Such prepared working solutions were used for the ABTS and DPPH assays. For that purpose, 10 µL of sample extract prepared according to the procedure described in Section 3.3.3, and 250 µL of five-times-diluted working solution (ABTS or DPPH) were dosed into 96-well plates. Incubation was carried out at room temperature (approximately 20 °C) in total darkness and lasted 6 and 30 min for ABTS and DPPH, respectively. After that, a plate reader (Multiskan Sky, Thermo Electron Co., Waltham, MA, USA) was used to measure the absorbance of prepared solutions at wavelengths set to 734 nm (ABTS assay) and 515 nm (DPPH assay), in relation to the blank test (10 µL of solvent instead of 10 µL of sample extract). The level of radical scavenging was presented as the EC_50_ coefficient—the sample extract concentration required for a 50% reduction in the initial amount of ABTS^•+^ radical cations and DPPH^•^ radicals—in mg of dry matter (d.m.) in 100 mL of sample extract. Each sample was tested four times (two sample extracts analyzed twice).

#### 3.3.6. Thermal Analyses

##### Thermogravimetric Analysis (TGA)

Thermogravimetric analysis was accomplished with the usage of the thermal analyzer (TGA/DSC 3+, STAR^®^ System, Mettler Toledo, Greifensee, Switzerland) to assess the thermal stability and decomposition of the compounds contained in the obtained dried strawberries. For that purpose, approx. 8.4 mg of a piece cut from a dried slice of strawberry was put into the 70 µL Al_2_O_3_ crucible, and it was subjected to heating from 30 to 600 °C under the nitrogen atmosphere. The heating rate and N_2_ flow were equal to 5 °C/min and 50 mL/min, respectively. Both TG and DTG curves—thermograms obtained using STAR^®^ Evaluation Software (version 16.10, Mettler Toledo, Greifensee, Switzerland)—were acquired from the differential values of the dependence of weight [%] on temperature and derivative weight [%/°C] on temperature, respectively.

##### Differential Scanning Calorimetry (DSC)

Differential scanning calorimetry was performed using a calorimeter (DSC 3+, STAR^®^ System, Mettler Toledo, Greifensee, Switzerland). In order to generate DSC thermograms (STAR^®^ Evaluation Software, version 16.10, Mettler Toledo, Greifensee, Switzerland) with the appointed glass transition temperature (T_g_), approx. 5.4 mg of a piece cut from a dried slice of strawberry was put into the 50 μL hermetic aluminum pan, and it was subjected to cooling from 25 to −50 °C, and after spending 5 min at the lowest temperature, it was heated from −50 to 150 °C. The entire process was carried out under the nitrogen atmosphere. The cooling rate, the heating rate, and the N_2_ flow were equal to −8 °C/min, 8 °C/min, and 50 mL/min, respectively.

#### 3.3.7. Fourier-Transform Infrared Spectroscopy (FTIR)

Fourier-transform infrared spectroscopy was executed in order to evaluate the chemical composition and the molecular structure of the obtained dried strawberries. Infrared spectra for the unground materials were recorded with the usage of the Cary 630 Spectrometer with MicroLab FTIR Software in version 5.7 (Agilent Technologies Inc., Santa Clara, CA, USA) in the wavenumber range of 4000–650 [1/cm], at the resolution of 4 [1/cm], and the number of scans equaled 32. A diamond was used as an attenuated total reflectance (ATR) sensor with a single reflection interface.

#### 3.3.8. Statistical Analysis

The obtained results were statistically analyzed via one-way analysis of variance (ANOVA) with Tukey’s test (Statistica, version 13, TIBCO Software Inc., Palo Alto, CA, USA) at a significance level of *p* < 0.05.

## 4. Conclusions

The conducted research proves that the pulsed electric fields (PEF) treatment and type of drying method do in fact affect the chemical and thermal characteristics of the obtained dried strawberries. The impact of PEF (e.g., by releasing substrates of enzymatic reactions and enzymes themselves) on the content of various sugars and antioxidant compounds has been demonstrated. The most important finding relates to changes in the share of individual sugars (sucrose, glucose, and fructose) in the total sugar content (TSC) in dried strawberries. Compared to untreated samples, PEF-pretreated samples exhibited a much lower share of sucrose in TSC, but at the same time a higher (up to 45%) share of glucose and fructose, which may point to the induction of sucrose inversion by PEF. Reduced exposure to oxygen and decreased drying temperature during vacuum drying led to obtaining dried strawberries with the highest content of antioxidant compounds, which are sensitive to these factors. All generated TG/DTG curves for dried strawberries exhibited a similar pattern, with five stages of thermal decomposition. The glass transition (T_g_) of all samples occurred at temperatures ranging from −1.2 to 14 °C. However, the PEF-pretreated dried strawberries had a lower glass transition temperature than the untreated samples, which confirms the increased mobility of the system after the application of an electric field. This indicates that these samples were more susceptible to physicochemical changes and microbial growth. All samples showed a similar pattern of IR spectra, with slight differences in the intensity of the obtained peaks. Therefore, it can be concluded that these samples consisted of chemical compounds with similar functional groups but with different concentrations (contents) of particular molecular bonds.

## Figures and Tables

**Table 1:** Data obtained from TGA and DSC analyses for untreated and PEF-pretreated strawberries dried by various methods (CD—convective drying; IR-CD—infrared-convective drying; MW-CD—microwave-convective drying; VD—vacuum drying).

Sample	Step 1		Step 2		Step 3		Step 4		Step 5		Sum [%]	T_g_ [°C]
	Temp. Range [°C]	Weight Loss [%]	Temp. Range [°C]	Weight Loss [%]	Temp. Range [°C]	Weight Loss [%]	Temp. Range [°C]	Weight Loss [%]	Temp. Range [°C]	Weight Loss [%]		
CD70	30–110	2.9	110–165	12.5	165–250	27.2	250–380	17.6	380–600	8.8	69.0	14.0
CD70_PEF1	30–110	3.2	110–165	13.9	165–250	24.2	250–380	17.3	380–600	8.7	67.3	0.1
CD70_PEF4	30–110	3.4	110–165	16.2	165–250	18.2	250–380	18.8	380–600	9.1	65.8	1.6
IR-CD20	30–110	3.3	110–165	14.6	165–250	25.9	250–380	17.9	380–600	8.8	70.5	10.2
IR-CD20_PEF1	30–110	6.1	110–165	12.6	165–250	24.8	250–380	18.9	380–600	9.2	71.5	13.9
IR-CD20_PEF4	30–110	3.4	110–165	16.2	165–250	21.7	250–380	18.1	380–600	9.2	68.8	14.1
MW-CD200	30–110	2.8	110–165	18.4	165–250	19.3	250–380	17.8	380–600	8.9	67.1	4.0
MW-CD200_PEF1	30–110	2.6	110–165	16.3	165–250	22.8	250–380	16.8	380–600	9.0	67.6	−1.2
MW-CD200_PEF4	30–110	3.8	110–165	14.4	165–250	25.5	250–380	17.2	380–600	8.7	69.6	0.8
VD55	30–110	2.5	110–165	15.4	165–250	23.8	250–380	19.3	380–600	9.5	70.5	7.0
VD55_PEF1	30–110	3.4	110–165	13.8	165–250	23.1	250–380	18.9	380–600	9.1	68.1	0.9
VD55_PEF4	30–110	3.1	110–165	7.3	165–250	30.1	250–380	20.2	380–600	10.2	71.0	4.2

## Data Availability

The data are contained within the article.
